# Smokers’ and drinkers’ choice of smartphone applications and expectations of engagement: a think aloud and interview study

**DOI:** 10.1186/s12911-017-0422-8

**Published:** 2017-02-28

**Authors:** Olga Perski, Ann Blandford, Harveen Kaur Ubhi, Robert West, Susan Michie

**Affiliations:** 1 Department of Clinical, Educational & Health Psychology, University College London, 1-19 Torrington Place, London, WC1E 6BT UK; 2 UCL Interaction Centre, University College London, 66-72 Gower Street, London, WC1E 6EA UK; 3 Department of Epidemiology and Public Health, Cancer Research UK Health Behaviour Research Centre, University College London, 1-19 Torrington Place, London, WC1E 6BT UK

**Keywords:** Alcohol reduction, Behaviour change, Engagement, Excessive alcohol consumption, mHealth, Smartphone apps, Smoking cessation, Think aloud, Thematic analysis

## Abstract

**Background:**

Public health organisations such as the National Health Service in the United Kingdom and the National Institutes of Health in the United States provide access to online libraries of publicly endorsed smartphone applications (apps); however, there is little evidence that users rely on this guidance. Rather, one of the most common methods of finding new apps is to search an online store. As hundreds of smoking cessation and alcohol-related apps are currently available on the market, smokers and drinkers must actively choose which app to download prior to engaging with it. The influences on this choice are yet to be identified. This study aimed to investigate 1) design features that shape users’ choice of smoking cessation or alcohol reduction apps, and 2) design features judged to be important for engagement.

**Methods:**

Adult smokers (*n* = 10) and drinkers (*n* = 10) interested in using an app to quit/cut down were asked to search an online store to identify and explore a smoking cessation or alcohol reduction app of their choice whilst thinking aloud. Semi-structured interview techniques were used to allow participants to elaborate on their statements. An interpretivist theoretical framework informed the analysis. Verbal reports were audio recorded, transcribed verbatim and analysed using inductive thematic analysis.

**Results:**

Participants chose apps based on their immediate look and feel, quality as judged by others’ ratings and brand recognition (‘social proof’), and titles judged to be realistic and relevant. Monitoring and feedback, goal setting, rewards and prompts were identified as important for engagement, fostering motivation and autonomy. Tailoring of content, a non-judgmental communication style, privacy and accuracy were viewed as important for engagement, fostering a sense of personal relevance and trust. Sharing progress on social media and the use of craving management techniques in social settings were judged not to be engaging because of concerns about others’ negative reactions.

**Conclusions:**

Choice of a smoking cessation or alcohol reduction app may be influenced by its immediate look and feel, ‘social proof’ and titles that appear realistic. Design features that enhance motivation, autonomy, personal relevance and credibility may be important for engagement.

**Electronic supplementary material:**

The online version of this article (doi:10.1186/s12911-017-0422-8) contains supplementary material, which is available to authorized users.

## Background

Cigarette smoking and excessive alcohol consumption are two of the most serious global public health problems [1]. Behaviour change interventions delivered face-to-face by trained healthcare professionals have been developed to help tackle them [2, 3]. With technological developments, behavioural interventions can now be delivered remotely via digital platforms. Digital behaviour change interventions include any behaviour change programme delivered via websites, mobile phones, smartphone applications (apps) or wearables [4]. Smartphones are typically carried with the user throughout the day and can therefore facilitate the delivery of behavioural support “just-in-time”, independent of geographical location [5–7]. Although only a minority of available smoking cessation and alcohol reduction apps have been rigorously evaluated in, for example, randomised controlled trials (RCTs), preliminary results suggest that apps might be effective in supporting smokers to quit and excessive drinkers to reduce their alcohol consumption [8–12]. In order to benefit from smoking cessation and alcohol reduction apps, users must identify and select which apps to download from the myriad available on the market [13, 14] and engage with them over time [15]. To our knowledge, no study has yet explored what factors are important in shaping this selection and subsequent engagement.

Although public health organisations such as the National Health Service in the United Kingdom (UK) and the National Institutes of Health in the United States (US) provide access to online libraries of publicly endorsed health apps (e.g. https://www.nhs.uk/oneyou/apps; https://www.nlm.nih.gov/mobile/) [16, 17], the majority of these accredited apps fail to act in accordance with data protection principles, such as encrypting personal information transmitted to developer or third-party servers [18]. There is also little evidence to suggest that users rely on these online libraries when searching for and selecting novel apps. Rather, the two most frequently used methods of identifying new apps are to search an online store and to seek recommendations from friends and family [19]. As there are currently more than 400 smoking cessation and 700 alcohol-related apps available on the market [13, 14], the onus is on the user to actively select which app to download. Notwithstanding a recent increase in the development and formal evaluation of theory- and evidence-informed apps within the research community [8–11, 20, 21], the majority of popular smoking cessation and alcohol reduction apps do not include ‘behaviour change techniques’ associated with higher quitting rates in face-to-face interventions and do not adhere to public health guidelines [13, 14, 22–26].

While popular smoking cessation and alcohol reduction apps vary in their specific approaches to behaviour change, commonalities in the techniques employed have been identified. For example, four independent content analyses of smoking cessation apps available in the US [13, 24], UK [23] and South Korean [26] versions of the iTunes Store/Google Play Store found that at least one of the following techniques was employed in a large proportion of the reviewed apps: self-monitoring (e.g. tracking cigarettes smoked or days smoke-free), feedback on progress, advising on how to quit, rewarding abstinence, supporting identity change and hypnosis [13, 23, 26]. Three independent content analyses of alcohol-related apps available in the US [25], Australian, [22] and UK [14] versions of the iTunes Store/Google Play Store found that although the majority of apps actively encouraged alcohol consumption, those promoting alcohol reduction commonly employed at least one of the following techniques: self-monitoring, feedback on progress (e.g. money saved from not buying alcohol), social support (e.g. dialling one’s sponsor), psychoeducation (e.g. information on the negative effects of excessive alcohol use) and hypnosis (e.g. audio recordings to encourage relaxation) [14, 22, 25]. With regards to features aimed at promoting engagement, one review of smoking cessation apps found that tailoring of content was employed in 45% of apps [24] while another review identified a decline in the use of engagement features such as tailoring of content and rewards (e.g. points/badges) in smoking cessation apps between 2012 and 2014 (69.6% reducing to 45.3%) [23].

Due to the variable quality of available smoking cessation and alcohol reduction apps, an important goal is to determine how the design of evidence-based apps can be improved to attract users’ attention in online stores and hence, increase their likelihood of being selected and engaged with [27]. The choice of any kind of app in an online store is likely to be influenced by visceral reactions to the app’s design and affective responses to and cognitive processing of the app’s known attributes [28–31]. Lasting positive first impressions of the visual appeal of websites are formed rapidly (within 50–500 milliseconds of exposure) and are primarily based on affective responses [28, 29]. While visual appeal was highlighted by users as important when choosing from pre-specified lists of apps (e.g. health apps, games for entertainment), factors such as perceived usefulness, personal relevance, positive user ratings and prior knowledge of brand names were also considered vital [30, 31]. There appears to be a lack of evidence as to how users freely choose smoking cessation and alcohol reduction apps in an online store and what factors shape their choice.

The potential benefits of apps depend not only on good choices by users but also on their subsequent engagement [15]. A positive association between engagement and intervention effectiveness has been observed [32, 33], suggesting that the likelihood of successful behaviour change depends on engagement with the intervention [15, 34]. In the context of digital behaviour change interventions, engagement has been defined as 1) the extent of intervention use (i.e. amount, depth, duration, frequency), and 2) a subjective experience characterised by attention, interest and affect [35]. Although it is unclear what level of engagement is required for different kinds of digital behaviour change interventions to be effective, engagement with health apps has typically been found to be low; it has been estimated that 25% of such apps are not used more than once by each user and that less than 10% of users return seven days after their first use [36, 37]. It is therefore important to identify design factors that promote or detract from engagement with digital health products [38].

Although numerous intervention studies have identified demographic (e.g. age, gender, educational attainment) and psychological (e.g. motivation, mental health status) factors that predict engagement with digital behaviour change interventions, few studies have employed experimental designs to evaluate the effect of specific design features on engagement (see [35] for a systematic review of 117 articles). While evidence from RCTs indicates that features such as reminders and prompts [39], tailoring of content [40], contact with a healthcare professional [41] and simultaneous delivery of content (as opposed to sequential delivery) [42] positively influence engagement with computer- and web-delivered behaviour change interventions, little is known about the specific design features that influence engagement with smoking cessation and alcohol reduction apps.

Results from a secondary analysis of automatically recorded usage data from an RCT of a smoking cessation app indicated that users more frequently engaged with some tools compared with others (i.e. ‘developing a quit plan’, ‘tracking smoking’, ‘viewing progress’) [43]; however, the effect of particular design features (e.g. ease of use, tailoring of content, rewards) on engagement was not explored. In a formal consensus exercise, behaviour change and alcohol experts rated features such as ease of use, tailoring of content, feedback, aesthetic appeal and ‘unique smartphone features’ as likely to engage users with a novel alcohol reduction app [44]; however, it is unclear whether experts’ views align with those of users from the target population. A cross-sectional survey of users’ views on the functionality of an alcohol reduction app developed based on guidance from the National Institute of Clinical Excellence found that users largely held favourable views towards the app’s features (e.g. an alcohol tracker, information on excessive alcohol use, notifications) [45]; however, users from the target population were not involved in the design of the app and survey respondents were not prompted to reflect on how the app’s features might influence their engagement. A qualitative study that explored young adults’ views on behaviour change apps and what factors contribute to their willingness to engage with such apps found that accuracy, security and immediate effects on mood were considered important for engagement while context-sensing software features and sharing on social media were considered off-putting [46]. However, no study to date has explored smokers’ and drinkers’ views on what design features are likely to be important for engagement with smoking cessation and alcohol reduction apps.

To better guide the selection of design features that can be included in future experimental studies (e.g. factorial RCTs), it would be useful to identify design features that smokers and drinkers judge to be important for engagement with smoking cessation and alcohol reduction apps. The present study therefore aimed to address the following two research questions through the use of qualitative methods:

1. What design features shape smokers’ and drinkers’ choice of smoking cessation and alcohol reduction apps?
2. What design features are judged by potential users to be important for engagement with smoking cessation and alcohol reduction apps?

## Methods

### Study design

The Consolidated Criteria for Reporting Qualitative Research checklist was used in the design and reporting of this study [47]. A think aloud methodology was used to address the first research question, which involved asking participants to verbalise their thoughts, impressions and feelings whilst engaging with an app of their choice [48]. The role of the researcher in a think aloud study is to retreat to the background and only prompt participants when necessary. This method was chosen over a retrospective design due to its ability to generate real-time data on the selections made, which was considered more reliable than data generated from participants’ memory. Semi-structured interview techniques were used to allow participants to elaborate on statements made during the think aloud tasks and to address the second research question. Behaviour is often influenced by unconscious processing of stimuli [49], so users may have limited insight into the factors that in fact influence their engagement with apps. However, user-centred design methods emphasise the importance of exploring users’ views as part of the iterative design process in order to develop digital behaviour change interventions that accommodate the needs of the target population [50–52].

### Theoretical framework

As we were interested in exploring novel themes not previously identified in the literature, an interpretivist theoretical framework was used to inform data gathering and analysis [53]. Interpretivism proposes that multiple realities exist (i.e. assumes a ‘subjective’ rather than ‘objective’ reality) and that participants’ accounts of their “lived experience” are co-constructed through the interaction with and subsequent interpretations of the researcher [53, 54]. Interpretivism recognises the active role of the researcher in both the elicitation and interpretation of qualitative data.

### Participants

Smokers were eligible to take part if they i) were aged ≥ 18 years, ii) smoked cigarettes daily, iii) would consider using a smartphone app to help them stop smoking, iv) owned an Android or iOS smartphone with internet access that was capable of running apps and v) lived in or near London (UK). Drinkers were eligible to participate if they i) were aged ≥ 18 years, ii) reported an Alcohol Use Disorders Identification Test-Consumption (AUDIT-C) score ≥ 5, indicating excessive alcohol consumption [55], iii) would consider using a smartphone app to help them reduce their drinking, iv) owned an Android or iOS smartphone with internet access that was capable of running apps and v) lived in or near London (UK). Smokers and drinkers interested in using an app to stop or cut down were recruited in order to mimic real-world conditions and hence generate more valid data. It was expected that these participants would be able to more vividly imagine engaging with the apps compared with smokers and drinkers uninterested in using an app to stop or cut down [56]. For pragmatic reasons, no cut-off was imposed on cigarettes per day for including smokers in the study. As approximately 47% of English smokers are interested in using a digital intervention to stop [57], it was deemed more important to recruit smokers who were interested in using an app to stop rather than heavy or highly dependent smokers. Participants who were both smokers and drinkers were only asked about one kind of app; they were allowed to indicate a preference for what behaviour to focus on. Participants who had already tried to quit smoking/reduce their drinking using an app were not excluded. Participants who were not fluent English speakers were excluded.

### Sampling

Participants were recruited through social media (e.g. Facebook, Twitter) and posters placed on central London university campuses. The recruitment materials stated that smokers and drinkers were invited to the laboratory to complete a few smartphone-based tasks and share their views on smoking cessation or alcohol reduction apps. Snowballing techniques were also used by asking participants to refer friends or family members interested in using an app to stop smoking or cut down on drinking [58]. Participants were recruited in batches of five until theoretical saturation was judged to have occurred (i.e. when no novel themes were identified) [59]. Preliminary data analysis was conducted after each batch of five participants to determine if more participants were needed.

### Measures

Data were collected at baseline on: 1) age; 2) gender; 3) ethnicity, measured using the Office for National Statistics’ index [60]; 4) socio-economic status, measured using the self-reported version of the National Statistics Socio-Economic Classification [61]; 5) nicotine dependence, measured using the Heaviness of Smoking Index (HSI) [62, 63]; a score ≥ 4 on the HSI indicates high nicotine dependence [63]; 6) patterns of alcohol consumption, measured using the Alcohol Use Disorder Identification Test-Consumption (AUDIT-C) [55, 64, 65]; an AUDIT-C score ≥ 5 indicates excessive alcohol consumption [55]; 7) motivation to stop smoking or cutting down on drinking, measured using the Motivation To Stop Scale (MTSS) [66]; 8) whether they had tried to stop/cut down in the past 12 months; 9) whether they had ever used an app to stop smoking/reduce drinking; 10) frequency of app use; 11) last time they had downloaded an app.

### Procedure

Participants read the information sheet which described the nature of the study without disclosing information that might have influenced participants’ search behaviours or verbal responses. They subsequently provided informed consent using an online screening questionnaire that assessed study eligibility and collected descriptive data (see Additional file 1). This questionnaire was hosted by Qualtrics survey software [67]. The face-to-face sessions were conducted in a private space at a London university or in participants’ homes, according to participant preference. No one else was present besides the participant and researcher except for one interview that was conducted in a space where university students were present. Interviews took place between April and June 2016. Sessions lasted between 45 and 75 min. Participants received a £20 gift voucher as compensation for their time.

#### Pre-task interview

A pre-session interview was held to elicit participants’ expectations of apps in general and smoking cessation or alcohol reduction apps in particular (see Additional file 2). Knowledge of participants’ existing beliefs about apps and their smoking/drinking identity was judged to be relevant for the interpretation of subsequent statements and reactions; for example, knowledge that a participant did not identify as an excessive drinker was subsequently used to interpret ambiguous statements or reactions to the explored apps.

#### Think aloud tasks

Participants were instructed on how to think aloud (see Additional file 2) and were subsequently asked to complete a practice task: thinking aloud whilst changing the ringtone on their smartphone. Participants were then asked to complete two tasks on their smartphone. The first involved searching for smoking cessation or alcohol reduction apps in an online app store and was designed to elicit thoughts about factors that shape smokers’ and drinkers’ decisions to download such apps. The second task involved downloading and exploring a free smoking cessation or alcohol reduction app and was designed to gain insight into factors expected to be important for engagement (see Additional file 2). Positive reinforcement was used to ensure that participants verbalised relevant information (e.g. “You’re doing well!”). When participants fell silent, prompts were used (e.g. “What are you thinking now?”).

#### Debrief interview

The purpose of the debrief interview was to give participants the opportunity to elaborate on statements made during the think aloud tasks. Following the analysis of the first two batches of interview transcripts, the semi-structured interview schedule was adapted in order to elicit more data about points raised by the first 10 participants (see Additional file 2). At the end of the sessions, participants were told the full purpose of the study.

### Data analysis

Sessions were audio-recorded, transcribed verbatim and analysed using inductive thematic analysis [68], which has previously been used to analyse data from think aloud studies involving smartphone apps [46, 69]. Braun and Clarke identify six phases of thematic analysis: i) familiarising with the data, ii) generating initial codes, iii) searching for themes, iv) reviewing themes, v) defining and naming themes, and vi) producing the report [68]. Data were coded by the first author using NVivo 10 [70] with regular discussions with the second author. New inductive codes were labelled as they were identified during the coding process. Data were sometimes assigned to multiple codes. All codes that potentially included data relating to the study aims were recorded. The first author reviewed the codes one by one, ordering the findings systematically under headings. The ordered data were reviewed and revised in discussion with the second author and were subsequently organised into themes. Theoretical saturation was judged to have occurred after 20 participants, as no new themes were identified [59]. As a quality check, the third author reviewed the codes, themes and participant quotes. Disagreements were resolved through discussion. Agreement on the final themes was reached through discussion between all co-authors. Differences between smokers and drinkers and other group differences were recorded where identified.

### External validation

Respondent validation refers to the comparison of the researcher’s interpretation of the data with participants’ accounts to assess the level of agreement between the two [71, 72]. A subsample of five participants (25%) was contacted and asked to review the results after the initial themes had been developed. Participants were asked to comment on whether they felt that their views were well represented and the extent to which they agreed with the interpretation of their quotes and the main claims of the narrative. Three participants returned their comments, stating that they agreed with the authors’ interpretations.

### Reflexivity

Despite smoking and excessive drinking being associated with social stigma [73, 74], the interviewer felt that good rapport was built with the majority of participants. At the beginning of the study, the interviewer asked each participant the same set of questions in the same order, but it later became apparent that a more discursive style generated more extensive data and was therefore adopted.

### Ethical approval

University College London’s Departmental Research Ethics Committee granted ethical permission (UCLIC/1213/015). Personal identifiers were removed from the data, which were stored securely, and principles of research governance were observed [75].

## Results

### Participant characteristics

The average age of participants was 29.7 years (*SD* = 9.2), 60% were women, 70% were of White ethnicity, 20% were of Asian ethnicity, 85% were from a high socio-economic status background and 55% of participants had made an attempt to quit smoking or cut down on their drinking in the past 12 months but had relapsed into smoking/drinking (i.e. all participants were smoking/drinking at the time of the study). Smokers had an average HSI score of 0.6 (*SD* = 1.07), indicating low nicotine dependence, and drinkers had an average AUDIT-C score of 7.0 (*SD* = 2.9), indicating excessive alcohol consumption. Participant characteristics are found in Table 1.

**Table 1:** Participants’ demographic, smoking, and drinking characteristics

ID	Group	Gender	Age	MTSS^a^	Made an attempt to stop/cut down in past 12 months	Ever used app to stop smoking or reduce drinking	Last time downloaded a smartphone app	Frequency of app use
D1	Drinker	M	24	5	Yes	No	In the last week	Daily
D2	Drinker	M	28	2	No	No	Today or yesterday	Daily
D3	Drinker	F	28	3	Yes	No	In the last month	Daily
D4	Drinker	F	31	6	No	No	In the last month	Weekly
D5	Drinker	F	21	2	No	No	Today or yesterday	Daily
D6	Drinker	F	56	2	No	No	In the last 6 months	Monthly
D7	Drinker	F	25	2	No	No	In the last 6 months	Daily
D8	Drinker	M	24	3	Yes	No	In the last month	Daily
D9	Drinker	M	47	3	Yes	No	In the last week	Daily
D10	Drinker	M	29	5	Yes	No	In the last week	Daily
S1	Smoker	M	24	2	No	No	In the last month	Several times/week
S2	Smoker	F	25	4	Yes	No	In the last week	Daily
S3	Smoker	M	28	3	No	No	In the last week	Daily
S4	Smoker	F	20	4	Yes	Yes	Today or yesterday	Daily
S5	Smoker	F	25	5	Yes	Yes	In the last week	Daily
S6	Smoker	F	27	7	Yes	No	In the last 3 months	Daily
S7	Smoker	M	25	2	No	No	In the last month	Daily
S8	Smoker	F	45	7	Yes	No	In the last 6 months	Daily
S9	Smoker	F	33	2	No	No	In the last week	Daily
S10	Smoker	F	28	5	Yes	No	In the last 3 months	Several times/week

^a^Motivation To Stop Scale (MTSS): 1 = I don’t want to stop smoking/cut down on drinking alcohol, 2 = I think I should stop smoking/cut down on drinking alcohol but I don’t really want to, 3 = I want to stop/cut down but haven’t thought about when, 4 = I really want to stop/cut down but I don’t know when I will, 5 = I want to stop/cut down and hope to soon, 6 = I really want to stop/cut down and intend to in the next 3 months, 7 = I really want to stop/cut down and intend to in the next month

### Themes

Three themes were developed in relation to the first research question and were labelled “immediate look and feel of the app”, “social proof” and “realistic and relevant titles”. Five themes were developed in relation to the second research question and were labelled: “features that enhance motivation”, “features that enhance autonomy”, “features that enhance personal relevance”, “features that enhance credibility” and “consistency with online and offline social preferences”. As few differences between smokers and drinkers were identified, groups were combined for the reporting of the results unless otherwise stated. A summary of the identified themes is found in Table 2. Supplementary excerpts from the face-to-face sessions can be found in Additional file 3.

**Table 2:** Summary of identified themes

	Theme	Description
1. What design features shape smokers’ and drinkers’ choice of apps?	The immediate look and feel of the app	First impressions of the app’s aesthetic appeal (e.g. colour scheme, minimalist design) and usability (e.g. easy to understand, not too text-heavy).
	Social proof	The app’s perceived quality, largely determined by ‘social proof’ (i.e. other users’ ratings, recognition of credible brands/institutions).
	Realistic and relevant titles	Titles that appeared realistic and relevant to the target behaviour (e.g. “quit smoking”, “reduce your drinking”).
2. What design features are judged to be important for engagement?	Features that enhance motivation	Features that enhanced participants’ motivation to stay smoke-free/reduce their drinking (e.g. monitoring and feedback, goal setting, rewards).
	Features that enhance autonomy	Features that enhanced participants’ autonomy (e.g. user-controlled reminders, flexible quitting/reduction plans).
	Features that enhance personal relevance	Features that engendered a sense of personal relevance (e.g. tailoring of content, a non-judgmental communication style, gain-framed messages).
	Features that enhance credibility	Features that engendered a sense of credibility and trust (e.g. a clear privacy policy, information perceived to be accurate).
	Consistency with online and offline social preferences	Consistency with participants’ attitudes towards sharing progress on social media or joining an online support community (i.e. online preferences) and their attitudes towards using the app to log cigarettes/units of alcohol or distract from cravings in social settings (i.e. offline preferences).

### What factors shape smokers’ and drinkers’ choice of apps?

#### The immediate look and feel of the app

The majority of participants (14/20) stated that their choice of apps was guided by the initial appeal of icons and screenshots; however, the specific factors contributing to judgments about attractiveness differed across participants. Half of the participants (10/20) mentioned feeling drawn to apps using bright colours (e.g. light green, white), which were described as attention-grabbing or associated with health and wellbeing, while apps using dark or neon colours were considered less appealing. This divide was not universal; a few participants (2/20) felt more drawn to apps in dark colours because these were perceived as taking the quitting process more seriously.

*Look at that! A dark screen, too many numbers. This really put me off. – D8*

When prompted to reflect on why particular designs caught their attention, many participants (9/20) mentioned that they preferred apps with minimalist or modern designs, as these were thought to signal professionalism and caring on the part of the developer, and described feeling “put off” by designs that looked “childish” or “amateurish”. However, the majority of participants (11/20) were unable to articulate exactly what they liked about a particular design. This was manifested by statements about the app simply looking “nice” or having the “right” look.

*Don’t like it, yeah. I can’t say more, it’s just intuitive, why. It’s just not something I’d particularly want to look at. - S8*

Many participants (9/20) mentioned that their choice was influenced by the app’s perceived usability or simplicity, as they did not wish to invest time in apps that seemingly required too much effort, appeared to be overly complex or evoked confusion.

*…they had these complicated graphs, and lots of information in your face, it would take you a while to read, whereas the app that I chose, it had information, it showed the progress, but it was much easier on the eye to read. - D1*

Judgments about an app’s ease of use were often interwoven with judgments about its aesthetic appeal (8/20), making it difficult to single out any one factor as being more important in guiding choice.

#### Social proof

The majority of participants (15/20) mentioned that taking other people’s star ratings or reviews of apps into account was vital in guiding their choice due to the lack of other guidance as to which apps are of acceptable quality. Choosing a popular app over a less popular one, determined by their respective number of downloads or list position, was thought to save time due to not having to manually filter out poor quality apps.

*…if an app has a good rating, despite the one or two people who are not satisfied, I think it would mean that it works for the majority of people. - S1*

Many participants (8/20) mentioned feeling drawn to apps from familiar brands, organisations or developers; these were described as being more salient than other apps. When prompted to reflect on why they felt drawn to familiar brands, participants stated that they expected such apps to be of better quality than those from unknown brands; they were uninterested in information provided by developers or organisations lacking authority.

*Who is […]? Whatever, I don’t care, you know. It’s just some guy who came up with an app. – S6*

#### Realistic and relevant titles

Many participants (9/20) mentioned that the app’s title was important in guiding their choice. Titles including key words such as “quit smoking” or “reduce your drinking” were considered appealing, as these appeared to provide a realistic summary of the app’s content. Participants avoided apps with titles that sounded like advertisements, such as those including the word “now”. These were thought to make empty promises about being able to help participants without providing any evidence for their statements. A few drinkers (3/10) avoided titles including the word “alcoholic”, as they did not believe that such apps would be personally relevant.

*I think the title is really, really important, in terms of, don’t give promises that… You’ve got to be really accurate and realistic, I think, to keep people interested. Don’t make claims like that, just easily. – S6*

### What factors are judged to be important for engagement?

#### Features that enhance motivation

The majority of participants (12/20) expected that regular monitoring of, for example, alcoholic beverages consumed or cigarettes smoked, and the receipt of feedback on their progress would be important for engagement. Being able to view a timeline of the days on which one had managed to stay smoke-free or drink less was expected to enhance motivation to continue, as participants did not want to “ruin their progress”.

*That’s probably a big incentive to not smoke, because it’s just going to set that back to zero, and it’s showing you your ever increasing progress, so yeah, I do like that. - S4*

Many participants (11/20) stated that they did not expect to re-engage with apps that were too difficult to use and/or confusing. A few participants (2/20) were particularly concerned that continuously opening the app to monitor their smoking or drinking would be too effortful and hence, lead to disengagement.

Many participants (8/20) mentioned that they expected goal setting to be engaging; they believed that the achievement of a goal would make them feel good about themselves and hence, increase their motivation to achieve further goals (i.e. a positive feedback loop).

*If you set those manageable goals, so you could achieve it, if you feel like you’re actually progressing, getting something, then you’re more likely to go back. - D10*

Of the 13 participants reacting to the provision of rewards within their selected app, approximately half (6/13) expected that the receipt of social or material rewards when achieving a goal, such as encouragement or badges, would increase their motivation to engage due to the desire to earn more rewards.

*Doesn’t [the badge] motivate you to carry on? You want to get more to prove to yourself that you can get them. – D5*

The other half of participants (7/13) were not convinced that earning virtual rewards would affect their motivation, as they did not attach any real value to intangible points or badges. A subtle difference between participants who had already tried to quit smoking or reduce their drinking in the past year and those who had not was observed; many (4/7) of those who had already tried to quit expressed negative attitudes towards the receipt of virtual rewards, perhaps suggesting that negative expectancy of such rewards might be linked to recent unsuccessful quit attempts.

*I’m not really going to get any awards, am I? They’re not giving me any money or presents. - D8*

#### Features that enhance autonomy

Of those expressing a desire to receive reminders to initiate engagement (11/20), the majority of these participants (9/11) wanted to control how frequently the app would contact them, as they had prior experiences of feeling bombarded or “bullied” by too many reminders.

*…it was getting really, really annoying, and it bullied me a little bit too much, about me not meeting my goals that I set in the beginning when I started using it. Then it just went the other way, and it just went out the door, and I just took it off my phone. - S3*

Many participants (9/20) already held firm beliefs about how to quit smoking or reduce their drinking. Smoking cessation apps that promoted a particular quitting strategy, such as quitting “cold turkey” with no option for gradual reduction, were therefore seen as inflexible. A few drinkers (4/10) expressed feeling annoyed with apps that rigidly compared their drinking patterns with the government’s recommended limits or persuaded users to have drink-free days, as they wanted to be in control of how to reduce their drinking in a meaningful way.

*…it seems a bit extreme, especially when you’re not an alcoholic, why do you need a drink free day? Can’t you just have a small glass of wine with your meal? – D7*

#### Features that enhance personal relevance

Tailoring of content according to individual preferences (13/20) inculcated a belief that the app was suited to the individual and that it was capable of providing effective support. For example, feedback on behavioural outcomes was estimated to be more engaging if it was tailored to the individual’s needs and preferences.

*I’m supposed to be motivated by how much money I’ve saved. That doesn’t make sense to me. I think I should be motivated by how my health might have improved. I don’t like this app. It’s not going to help me. - D6*

Information perceived as “preachy” or patronising made participants feel judged or nagged (9/20). This resulted in refusals to take the information seriously due to the desire to rebel against advice on what one “should” do.

*I think I’m more likely to listen to practical advice rather than finger wagging… - S9*

Some participants (6/20) mentioned that they wanted information about the positive effects of quitting or cutting down (i.e. ‘gain-framed’ messages). Information about health consequences that focused on the negative aspects of past smoking or drinking (i.e. ‘loss-framed’ messages) made participants (7/20) feel disempowered due to the inability to change past actions. Information focusing on the negative consequences of future smoking made some participants feel indifferent due to the inability to imagine one’s future self.

*Great. I started smoking when I was 13 and back then, I was smoking 40 cigarettes a day. - S3*

A few drinkers (3/10) were sensitive to terminology perceived as “serious” or harsh, especially when terms such as “alcoholic” or “addict” were used. They were quick to distance themselves from apps using such terminology, as they appeared to assume that these must be catered to individuals who, unlike them, were dependent on alcohol. Smokers were more accepting of the use of the term “addict”.

*“Add an addiction.” OK, quite serious… Wow! “I’ve been clean for…” That’s some serious terminology. - D10*

#### Features that enhance credibility

Many participants (8/20) mentioned that they felt uneasy about having to create an account with their personal e-mail address or allow access to the phone’s location services in order to use their selected apps, as they were worried that their information would be passed on to third parties.

*One thing is that I tend to not like apps that require so much data about my location services, because, I don’t know, but obviously they sell on apps, so I think I’m quite wary of telling people too much about my data… - S10*

However, a few participants (3/20) mentioned that their concerns were mitigated if a message about the app’s policy on privacy and confidentiality was provided due to feelings of trust. A few participants (2/20) explicitly stated that they had no concerns regarding privacy in the context of apps.

*It then says: “Your data will be anonymised and not shared with anybody other than for our research”, which is nice to tell people for confidentiality reasons. - D7*

Information judged to be inaccurate was met with scepticism by many participants (8/20) as errors and inconsistencies were thought to undermine the app’s credibility. Participants did not want to waste time on inaccurate advice, as this was deemed to be untrustworthy.

*I think it’s really important that these sorts of sites and apps have the most current, up-to-date information, in order to get me to trust them, and take on board what they’re telling me. - D2*

#### Consistency with online and offline social preferences

Of the participants who reacted to the provision of social support features within their selected apps (10/20), such as sharing progress on social media (e.g. Facebook, Twitter) or joining an online community, few (4/10) expressed a desire to engage with such features; smoking and drinking were seen as private behaviours that are unacceptable to share with one’s wider social network. Participants anticipated that sharing such information with others would generate pity rather than support.

*…what do I want to get from that? I’m not going to get endorsements, I’m just going to get a few sad likes that are going to be quite patronising to me… - S3*

A subtle difference was observed between those who had tried to quit smoking or reduce their drinking in the past year and those who had not; the former appeared to judge social sharing to not be engaging due to the anticipation of added pressure rather than increased support while the latter expressed more favourable attitudes towards social support features, especially those enabling users to join an online support community. Participants who had not made an attempt to quit expected that connecting with others in a similar situation might help stick to one’s goals due to increased motivation.

Beliefs about the capability of apps to provide timely support when experiencing a craving were mixed. Many participants (7/20) struggled to see ways in which engagement with an app would influence their waning resolve. A few smokers (3/10) believed that doing a breathing exercise to assuage cravings would be helpful in the moment, but they did not want to use distraction games when socialising with others, who might find this behaviour strange.

*Obviously, if you’re in a bar, you’re not going to be like: “I’m sorry guys, I just need to play my game.” Maybe when you’re home alone, it could be useful. – S5*

When imagining logging drinks consumed in social situations, a few drinkers (2/10) mentioned that they anticipated feeling embarrassed or uncomfortable, as others might find such behaviour “odd” or “rude” and hence, stop inviting them to the pub.

*If I pull it out and start pressing it every time I’ve had a drink, they’re going to start thinking that I’m odder than I really am. – D9*

## Discussion

This study found that the immediate look and feel of apps, social proof and realistic and relevant titles shape smokers’ and drinkers’ choice of apps. Features that enhance motivation, including monitoring and feedback, goal setting, ease of use and rewards, and those that enhance autonomy, including flexible prompts and quitting strategies, were judged to be important for engagement. Participants also expected that features that engender a sense of personal relevance, such as tailoring of content according to individual preferences and the use of a non-judgmental communication style, and those that engender a sense of credibility, including privacy and accuracy, would be engaging. Moreover, consistency with one’s online and offline social preferences was considered important for engagement. Few differences were found between smokers and drinkers.

The finding that the immediate look and feel of apps influenced participants’ choice is consistent with the argument that visceral reactions to an app’s design generate lasting positive first impressions [28, 29]. However, other people’s app ratings and the perceived relevance of titles were also considered important. This supports the suggestion that both affective responses and cognitive processing of an app’s attributes influence users’ choice of apps [30, 31].

Our results are consistent with a number of well-established findings. Firstly, the finding that prompts, rewards, ease of use and tailoring of content according to individual differences were expected to be important for engagement supports previous research into computer-delivered smoking cessation and alcohol reduction interventions [42, 76, 77], results from content analyses of smoking cessation apps [23, 24] and findings from a formal expert consensus study [44]. Secondly, the finding that the app’s communication style was judged to be important for engagement is consistent with previous research suggesting that the “tone of voice” of digital behaviour change interventions may evoke strong negative emotions and hence, cause participants to disengage [78]. Moreover, the finding that privacy and accuracy are expected to be important for engagement due to feelings of trust replicates research into other kinds of digital behaviour change interventions [79–81].

A frequently mentioned justification for using smartphone apps to deliver complex behaviour change interventions is that these are capable of delivering support as and when required, or “just-in-time” [5, 6]. As participants in the present study expressed concerns about engaging with smoking cessation and alcohol reduction apps in social settings due to anticipated embarrassment, this adds nuances to the assumption that smokers and drinkers want timely behavioural support irrespective of context. A recent study that employed geofencing (i.e. a software feature that uses the phone’s global positioning system to set up geographical boundaries) to deliver context-aware smoking cessation support found that only a small proportion of pre-quit smoking reports (6.1%) were logged in social situations [82]. One of the reasons for this, as evidenced in follow-up interviews with participants, was fear of appearing rude to other people. This finding is also consistent with views expressed by young adults in a qualitative study exploring opportunities and challenges for behaviour change apps, who questioned the accuracy of context-sensing features [46].

Consistent with previous findings [46], smokers and drinkers in the present study did not want to share progress with their wider social networks due to the belief that others would pity rather than encourage this. It has been found that so-called ‘closet’ quit attempts (i.e. attempts to stop smoking without disclosure to anyone) are common among smokers [83]. As non-disclosure does not appear to be associated with a decreased likelihood of cessation success [83], this may be interpreted to suggest that social sharing should not be considered a ‘one-size-fits-all’ approach.

Care should be taken not to overstate the importance of the present findings due to the subtle group differences observed and the small sample size. However, it was found that attitudes towards joining an online support community and attitudes towards the receipt of virtual rewards appeared to differ depending on whether participants had made an attempt to quit/cut down in the past year. This suggests that individuals may differ in the factors that influence their judgments of engagement features. Future research should explore whether individuals may respond differently to social support features and rewards depending on their demographic and/or psychological characteristics.

### Limitations

The method chosen to elicit data involved asking participants about their expectations about what factors would be engaging. As evidence suggests that the magnitude of relationships between beliefs and attitudes, intentions and actual behaviour are modest [84], further research is required to assess whether the inclusion of the features judged by participants to be important for engagement in the present study is in fact accompanied by higher levels of engagement. Although reliable methods for determining the potential of health apps to engage users (e.g. the Mobile Application Rating Scale [85]; a coding scheme developed by Ubhi and colleagues [86]) are available, the predictive validity of such scales (i.e. the scales’ ability to predict actual levels of engagement) has not been evaluated. As the purpose of the present study was to explore smokers’ and drinkers’ views of apps, consistent with a user-centred approach to intervention design [50–52], think aloud methodology and semi-structured interview techniques were deemed to be more appropriate than existing quality scales. It has been argued that the use of think aloud methodology to elicit data might be problematic as it is cognitively demanding for participants to complete the assigned tasks whilst verbalising their thoughts [87]. However, we attempted to mitigate this issue by conducting debrief interviews to allow participants to elaborate on their statements.

The boundary between aesthetic appeal and perceived usability was often unclear in participants’ explanations, highlighting the difficulty in articulating precisely why particular designs are considered more attractive than others and hence, indicating that the data generated here might be imprecise. However, ratings of beauty have been found to be strongly associated with ratings of perceived usability in other settings [88]. This emphasises the complexity of trying to dissociate these constructs and suggests that our findings are consistent with the published literature [28, 29]. Additional insight into how smokers and drinkers select apps (e.g. specific search terms used, non-conscious selection processes) might be gained from screen recordings or the use of eye tracking methodology.

As participants in the present study were predominantly of White ethnicity from high socio-economic status backgrounds and smokers indicated low levels of nicotine dependence it is possible that our findings do not generalise across the target population. However, participants reported similar levels of motivation to stop compared with a large, representative sample of English smokers (*N* = 2483): 35% in the present study versus 39% of English smokers in the earlier study indicated a MTSS score of ≥ 5 [66]. The finding that few smokers and none of the drinkers in the present study had ever used an app to quit smoking/reduce their alcohol consumption may be interpreted to suggest that the real concern is not how users decide which app to use, but rather, that it is more important to gain insight into what makes smokers and drinkers decide to use an app in the first place. Little is known about the uptake of smoking cessation and alcohol reduction apps in the general population of smokers and drinkers; however, findings from an ongoing series of cross-sectional household surveys of representative samples of the English population indicate that although half of smokers expressed an interest in using digital smoking cessation interventions (e.g. websites, smartphone apps), fewer than 1% had in fact used such interventions to support a quit attempt in the past year [57]. Hence, an alternative interpretation is that, according to available statistics, our sample appears similar to the target population with regards to previous app use.

### Implications and future directions

As smokers and drinkers tend to select apps at least partly based on their immediate look and feel, it is important for healthcare professionals to collaborate with interaction design experts to develop evidence-based smoking cessation and alcohol reduction apps that are on a par with other commercially available apps in terms of aesthetics and usability. As participants in the present study were found to rely on ‘social proof’ (i.e. other users’ ratings and brand recognition) when selecting apps, researchers and practitioners could leverage this by initiating collaborations with developers of popular apps or apps from well-known brands. For example, it might be more fruitful to modify the content of a well-established app with an existing client base rather than developing a novel smoking cessation or alcohol reduction app.

The finding that smokers and drinkers are more willing to engage with apps that provide options regarding quitting strategy poses a design challenge. As evidence suggests that some quitting strategies are more effective than others on average – for example, quitting smoking “cold turkey” is more effective than gradual reduction [89] – designers might benefit from using persuasive design elements, such as providing tutorials and guidance, using tunnelling techniques (i.e. making users click through a pre-specified sequence of pages), or making use of normative influence, to attempt to modify users’ beliefs and attitudes [90].

Our findings suggest that the specifics of how to personalise content to support smokers’ and drinkers’ needs to promote engagement merit further investigation. A data-driven approach using machine-learning techniques might be helpful in advancing the knowledge on how to meaningfully tailor app content according to individual differences. For example, the type of feedback provided or whether or not to offer features that link users with others on social media could be tailored according to individual preferences to foster a sense of personal relevance. Furthermore, smokers and drinkers expected that too many reminders would lead to habituation and reduce autonomy. One means of preventing this is to develop response-sensitive notifications. For example, daily notifications could be sent as long as users react to these but their frequency would be reduced, or timing changed, as soon as users stop reacting to the prompts.

The finding that few smokers and drinkers wanted to use the apps in social settings suggests that the social context in which cigarette and alcohol cravings are triggered (e.g. pubs, cafés) should be considered in the design process. Smoking and drinking are perceived as more private than, for example, physical activity behaviours, perhaps due to social stigma [73, 74]. It should therefore not be assumed that features included in apps targeting other types of behaviour can successfully be transferred to those targeting smoking and drinking. The hypothesis that smokers and drinkers might engage more with apps that suggest how to replace smoking and drinking with other activities as opposed to those that provide in-the-moment support could be tested in future research. See Table 3 for a summary of design recommendations.

**Table 3:** Summary of design recommendations

Category	Design Recommendations
How can the reach of evidence-based apps be improved?	Develop smoking cessation and alcohol reduction apps that are on a par with other commercially available apps in terms of aesthetics and usability, perhaps through collaboration with interaction design experts.
	Researchers and practitioners may consider initiating collaborations with developers of popular apps and/or apps from well-known brands to leverage their existing ‘social proof’.
	Use simple and straightforward titles that include key words (e.g. “quit smoking” or “reduce your drinking”).
How can engagement be improved?	Use persuasive design elements (e.g. guidance, tunnelling, normative influence) to modify users’ beliefs about how to quit smoking or reduce their drinking.
	Use machine-learning techniques to explore how to meaningfully tailor content according to individual differences (e.g. feedback, rewards).
	Develop response-sensitive notifications that tail off or adjust timings if the user stops reacting in order to prevent habituation or annoyance.
	Consider the online and offline social preferences of the target population. For example, it might be more fruitful to focus on action planning and/or behaviour substitution rather than in-the-moment support for smokers and drinkers.

## Conclusion

Smokers and drinkers interested in quitting or cutting down using a smartphone app choose apps based on their immediate look and feel, social proof and titles judged to be realistic and relevant. Features that enhance motivation, autonomy, personal relevance and credibility, and those that are consistent with users’ online and offline social preferences are rated by participants as important for engagement.

## Additional files

Additional file 1: Screening and baseline questionnaires. (DOCX 93 kb)
Additional file 2: Verbal instructions and semi-structured interview protocol. (DOCX 104 kb)
Additional file 3: Supplementary excerpts from the think aloud and interview sessions. (DOCX 110 kb)

## Abbreviations

AUDIT-C: Alcohol use disorders test-consumption
HSI: Heaviness of smoking index
MTSS: Motivation to stop scale
