# Supplementary material for: Smokers’ and drinkers’ choice of smartphone applications and expectations of engagement: a think aloud and interview study
Source: BMC Med Inform Decis Mak. 2017 Feb 28;17:25. doi: 10.1186/s12911-017-0422-8 (PMC5329928; doi:10.1186/s12911-017-0422-8)
Supplement: Additional file 1: — Screening and baseline questionnaires. (DOCX 93 kb) [file 12911_2017_422_MOESM1_ESM.docx]

**Additional file 1**

***Online screening questionnaire***

| **Question** | **Response Options** |
| --- | --- |
| How old are you? | Enter free text |
| Do you smoke cigarettes daily? | (1) Yes  (2) No |
| How often do you have a drink containing alcohol? | (0) Never  (1) Monthly or less  (2) 2 to 4 times a month  (3) 2 to 3 times a week  (4) 4 to 5 times a week  (5) 6 or more times a week |
| How many standard drinks containing alcohol do you have on a typical day when you are drinking? | (0) 1 to 2  (1) 3 to 4  (2) 5 to 6  (3) 7 to 9  (4) 10 to 12  (5) 13 to 15  (6) 16 or more |
| How often do you have six or more standard drinks on one occasion? | (0) Never  (1) Less than monthly  (2) Monthly  (3) Weekly  (4) Daily or almost daily |
| Do you live in or near London? | (1) Yes  (2) No |
| Do you own an iPhone or an Android smartphone with Internet access capable of running apps? | (1) Yes  (2) No |
| Would you consider using a smartphone app to help you stop/cut down on your smoking? | (1) Yes  (2) No |
| Would you consider using a smartphone app to help you cut down on your drinking? | (1) Yes  (2) No |

***Baseline questionnaire***

| **Question** | **Response Options** |
| --- | --- |
| What is your gender? | (1) Male  (2) Female |
| Do (did) you work as an employee or are (were) you self-employed? | (1) Employee  (2) Self- employed with employees  (3) Self-employed/freelance without employees |
| How many people work (worked) for your employer at the place where you work (worked)?  OR  How many people do (did) you employ? | (1) 1 to 24  (2) 25 or more |
| Do (did) you supervise any other employees? (A supervisor or foreman is responsible for overseeing the work of other employees on a day-to-day basis) | (1) Yes  (2) No |
| Please tick one box to show which best describes the sort of work you do. If you are not working now, please tick a box to show what you did in your last job. | (1) Modern professional occupations (teacher, nurse, physiotherapist, social worker, welfare officer, artist, musician, police office, software designer)  (2) Clerical and intermediate occupations (secretary, personal assistant, clerical worker, office clerk, call centre agent, nursing auxiliary, nursery nurse)  (3) Senior managers or administrators (finance manager, chief executive)  (4) Technical and craft occupations (motor mechanic, fitter, inspector, plumber, printer, tool maker, electrician, gardener, train driver)  (5) Semi-routine manual and service occupations (postal worker, machine operative, security guard, caretaker, farm worker, catering assistant, receptionist, sales assistant)  (6) Routine manual and service occupations (HGV driver, van driver, cleaner, porter, packer, sewing machinist, messenger, labourer, waiter/waitress, bar staff)  (7) Middle or junior managers (office manager, retail manager, bank manager, restaurant manager, warehouse manager, publican)  (8) Traditional professional occupations (solicitor, medical practitioner, scientist, civil/mechanical engineer) |
| What is your ethnic group? | (1) English/Welsh/Scottish/Northern Irish/British  (2) Irish  (3) Gypsy or Irish Traveller  (4) Any other White background  (5) White and Black Caribbean  (6) White and Black African  (7) White and Asian  (8) Any other Mixed/Multiple ethnic background  (9) Indian  (10) Pakistani  (11) Bangladeshi  (12) Chinese  (13) Any other Asian background  (14) African  (15) Caribbean  (16) Any other Black/African/Caribbean background  (17) Arab  (18) Any other ethnic group |
| Have you made an attempt to stop smoking in the past 12 months? | (1) Yes  (2) No |
| Which of the following best describes you? | (1) I don’t want to stop smoking  (2) I think I should stop smoking but I don’t really want to  (3) I want to stop smoking but I haven’t thought about when  (4) I REALLY want to stop smoking but I don’t know when I will  (5) I want to stop smoking and hope to soon  (6) I REALLY want to stop smoking and intend to in the next 3 months  (7) I REALLY want to stop smoking and intend to in the next month |
| Have you ever used a smartphone app to help you quit smoking? | (1) Yes  (2) No |
| How many cigarettes do you smoke per day? | Enter free text |
| How soon after waking do you usually smoke your first cigarette? | (0) 61+ minutes  (1) 31-60 minutes  (2) 6-30 minutes  (3) <5 minutes |
| How often did you experience urges to smoke in the past 24 hours? | 1. Not at all 2. A little of the time 3. Some of the time 4. A lot of the time 5. Almost always   (5) All the time |
| Have you made an attempt to cut down on drinking alcohol in the past 12 months? | (1) Yes  (2) No |
| Which of the following best describes you? | (1) I don't want to cut down on drinking alcohol  (2) I think I should cut down on drinking alcohol but don't really want to  (3) I want to cut down on drinking alcohol but haven't thought about when  (4) I REALLY want to cut down on drinking alcohol but I don't know when I will  (5) I want to cut down on drinking alcohol and hope to soon  (6) I REALLY want to cut down on drinking alcohol and intend to in the next 3 months  (7) I REALLY want to cut down on drinking alcohol and intend to in the next month |
| Have you ever used a smartphone app to help you cut down on your drinking? | (1) Yes  (2) No |
| How often did you experience urges to drink in the past 24 hours? | (0) Not at all  (1) A little of the time  (2) Some of the time  (3) A lot of the time  (4) Almost always  (5) All the time |
| When was the last time you downloaded an app, if ever? | (1) Today or yesterday  (2) In the last week  (3) In the last month  (4) In the last 3 months  (5) In the last 6 months  (6) More than 6 months ago |
| How frequently do you use the apps on your smartphone, if at all? | (1) Daily  (2) Weekly  (3) Monthly  (4) Never |
| Do your friends and family ask for your advice or help in using smartphone apps? | (1) Yes  (2) No |
| I use my smartphone to: | (1) Check my e-mail  (2) Find out what my friends are doing on Facebook  (3) Get information via Twitter  (4) Navigate using Google Maps or similar tools  (5) Read the news  (6) Research things to purchase  (7) Download and play games  (8) Download and use health/fitness apps |
