# Supplementary material for: Smokers’ and drinkers’ choice of smartphone applications and expectations of engagement: a think aloud and interview study
Source: BMC Med Inform Decis Mak. 2017 Feb 28;17:25. doi: 10.1186/s12911-017-0422-8 (PMC5329928; doi:10.1186/s12911-017-0422-8)
Supplement: Additional file 2: — Verbal instructions and semi-structured interview protocol. (DOCX 104 kb) [file 12911_2017_422_MOESM2_ESM.docx]

**Additional file 2**

***Pre-session interview***

1. Can you tell me about an app that you are using regularly? Why do you think that you are using it regularly?
2. Have you ever used a health or fitness app? Can you tell me about it?
3. What do you think a smoking cessation/alcohol reduction app should provide or do?

After the first half of the interviews, another question was added:

1. What is your identity as a smoker/drinker?

***Think aloud***

*Verbal instructions*

“During this session, you will be given two smartphone-based tasks to complete. I would like to emphasise that this is not a test; I am interested in the tasks themselves, not your performance. I would like you to complete the tasks whilst “thinking aloud”. This means that I would like you to complete the tasks, and while you do so, try to say everything that goes through your mind. I would like you to pretend that you are at home and try to forget that I am here.

Thinking aloud usually feels a bit strange at first, as it is an unusual task. Don’t worry about it, most people find it a bit unnatural at first, but quickly get used to it! We will start off with a practice task to make sure that you feel comfortable. I would like you to change the ring tone on your smartphone whilst trying to say everything that goes through your mind.”

*Tasks*

1. I would like you to imagine that you are at home. Please find an app that you think will be engaging enough to help you quit or cut down on your smoking/drinking. Please use the App Store/Google Play to search for apps whilst thinking aloud.
2. Imagine that you have selected a smoking cessation/alcohol reduction app that you would like to try. Please download one of the free apps that you think will be engaging enough to help you quit or cut down on your smoking/drinking. Please complete the baseline questions and explore the app whilst thinking aloud.

***Post-session interview***

1. I noticed that you mentioned that you thought that [...] was ... Can you tell me a bit more about that?
2. I noticed that you made a comment about [...]. Can you elaborate on that?
3. How do you understand the term “engagement” in the context of apps?
4. Do you think that the app that you chose to download was engaging? Why/why not?
5. Do you think that you would find the app/those particular features engaging longer term? Why/why not?
6. Do you think that you might use the app that you have downloaded after leaving this session? Why/why not?

After the first half of the interviews, the following questions were added:

1. You mentioned that you thought that [...] was ... How do you think that feature would fit into your daily life?
2. How important is it for you to be able to relate to the app’s content?
3. How do you think engaging with the app would help you stop/cut down on your smoking/drinking?
4. How do you think that [...] would help you stop/cut down on your smoking/drinking?
