# Supplementary material for: Smokers’ and drinkers’ choice of smartphone applications and expectations of engagement: a think aloud and interview study
Source: BMC Med Inform Decis Mak. 2017 Feb 28;17:25. doi: 10.1186/s12911-017-0422-8 (PMC5329928; doi:10.1186/s12911-017-0422-8)
Supplement: Additional file 3: — Supplementary excerpts from the think aloud and interview sessions. (DOCX 110 kb) [file 12911_2017_422_MOESM3_ESM.docx]

**Additional file 3.**

Supplementary excerpts from the think aloud and interview sessions.

| **Research Question** | **Theme** | **Example Excerpts** |
| --- | --- | --- |
| ***1. What factors shape smokers’ and drinkers’ choice of apps?*** | *The immediate look and feel of the app* | "...they were sort of dark and black and had some sort of neon lighting on it, and it just didn’t look very inviting, whereas that first one was actually really colourful and bright, and quite modern, and had illustrations…" – D3  "...it looks kind of inviting, with all the colours and exclamations and stuff." – D5  "I liked the logo because it was green, it looked minimalistic, so I thought that maybe the app will be easy to use, and not chaotic, just easy to use." – S5  "The pictures look very scientific, not very user-friendly at all." – D1  "It looks a lot more simple to use, and a simple user face." – S4 |
|  | *Social proof* | "It hasn’t had that many downloads, only 100, which seems quite low, not many people have used this." – D4  "None of these apps have any ratings, so it’s really hard to know what people are thinking of them, because I genuinely just go on the ratings." – S2  "So here’s the thing, this is what I normally do, I would just look at the stars, because other people have done this." – S6  "It’s also by […], so it just seems more trustworthy. I don’t know the other ones." – D7  "...the fact that it was by the […] made me want to go and look at it." – S2 |
|  | *Realistic and relevant titles* | "There’s one called […] but it’s got the word ‘alcoholic’ in it so I’m thinking it’s probably not for me." – D2  "“Dependence – quit tobacco and alcohol”. That sounds a bit hip, directed towards younger people than me." – D6  “Obviously, with that, maybe, I’d known what I was getting, which probably would have been more suitable, but, “Sober Time,” it didn’t seem like it was completely off topic, so it didn’t mislead me in that way.” – D10  "OK, this one is one of the condescending ones. “We are your motivation” kind of thing." – S3  “I think they could probably have chosen a better name. I didn’t like the whole “now” thing." – S6 |

| ***2. What factors are judged to be important for engagement?*** | *Features that enhance motivation* | "I liked that. That was a good idea. You have a goal to aim for, and then it’s saying: “You’ve reached it” or “You haven’t reached it.”" – D4  "...then you have these goals as well, and then obviously if you do have a cigarette then, you kind of ruin it…" – S5  "...it just tallies up how much you’re smoking, which is good, because just keeping track of something will automatically help you reduce…" – S2  "Rewards to keep me motivated to give all this information in the journal. Just to keep using it.” – S6  "I mean, I can see why these achievement things are useful, but for me, they don’t really bother me at all. I guess it’s trying to motivate you to get all these achievements, but for me, I just kind of don’t give a s*** about that." – D1 |
| --- | --- | --- |
|  | *Features that enhance autonomy* | "...if there’s an option saying: “How often do you want to be notified”, I think would be quite handy as well." – D4  "I want to be able to handle the app myself, and feel that it’s support for me, not taking over." – D6  "I like having control, but then, I probably will forget to use things unless it gives me a notification." – S9  "Because that 14 units of alcohol is just an irrelevance. It doesn’t apply to anyone at all." – D9  "I do want to quit cold turkey, and then I’d probably want one of those apps, but in the meantime, I would like an app that would also be for cutting down smoking, because that’s obviously helping too." – S5 |
|  | *Features that enhance personal relevance* | "...the app doesn’t personalise enough. Maybe in the registration bit, they could perhaps ask you what you’re interested in mostly." – D7  "“Heavy alcohol consumption is linked to a number of cancers, such as...” OK, thank you. Now you’re just scaring me. I don’t know if I like them telling me that." – D6  "...not only how you’re destroying everything with the smoking, because obviously that doesn’t go in, but maybe positive things will…" – S3  "It comes back to what I was saying about the guidelines coming across as preachey or whatever." – D1  "...it has very non-judgmental language in it, which is really good." – S10 |

|  | *Features that enhance credibility* | "Yeah, I’m not bothered about data concerns, or whatever, I’m quite happy to just give my information away." – D1  "I just don’t think I like to give my details to any random app that I’ve searched for, so, like I said, if someone recommended something to me, but yeah, I probably don’t like to give my information to anything more than I have to, and definitely not a random app with bad reviews..." – D3  "If you say something inaccurate, stupid, or lazy, you’re going to lose your credibility." – S6  "I don’t know whether they’ve just thought that could help or if they’ve actually conducted some research where people have said that this has helped, tips in the moment." – S9 |
| --- | --- | --- |
|  | *Consistency with online and offline social preferences* | "If I’m going to do this, it would be about what I was doing, I don’t really mind if there’s a strong community of people doing exactly the same thing.” – D10  "I don’t know, I don’t think I would want other people to know that I’m trying to reduce my drinking, personally." – D4  "I don’t see very many tips on that, the social aspect and how to get over the social aspect." – S10  "...that’s what I see as the psychology of quitting. If you are quitting by telling everyone that you’re quitting, it puts so much pressure on you." – S3  "“Post progress on Facebook or Twitter.” I wouldn’t be interested in that, I’m more of a private person." – S6 |
